# Epidemiology of Substance Use among University Students in Sudan

**DOI:** 10.1155/2016/2476164

**Published:** 2016-02-24

**Authors:** Tarig Osman, Cathrine Victor, Alaa Abdulmoneim, Hala Mohammed, Fatima Abdalla, Asma Ahmed, Eiman Ali, Wael Mohammed

**Affiliations:** Sudan Medical and Scientific Research Institute, Khartoum, Sudan

## Abstract

*Background*. Youth populations are vulnerable to substance use particularly in developing countries where circumstances may be favorable for it. There is no published data on substance use among the youth in Sudan other than on tobacco use.* Objectives*. The aim of this study was to investigate the prevalence, circumstances, and factors associated with substance use.* Methods*. An institution-based survey was conducted on a sample of 500 students. Data was collected using a questionnaire designed by the WHO for student drug surveys and analyzed using IBM SPSS version 20.* Results*. The overall prevalence of substance use is 31%. The current prevalence of tobacco, cannabis, alcohol, amphetamines, tranquilizers, inhalants, opiates, cocaine, and heroin use was 13.7%, 4.9%, 2.7%, 2.4%, 3.2%, 1%, 1.2%, 0.7%, and 0.5%, respectively. Curiosity (33.1%) was the main reason for initiation of substance use. The main adverse effects reported were health problems (19.7%) and theft (19.7%). Peers (40.9%) were the prime source of substance use. On multivariate analysis, male sex was the principle predictor for substance use (AOR: 5.55; 95% CI: 3.38, 9.17).* Conclusion*. Strategies to control substance use should encompass the role of the university and parents in observing and providing education to improve awareness of substances and their consequences.

## 1. Introduction 

Substance use is a major global public health issue [1]. In 2011, it was estimated that between 167 and 315 million people or 3.6%–6.9% of the adult population aged 15 years to 64 years have used an illicit substance in the preceding year [2]. The global disease burden attributable to alcohol and illicit drugs is estimated at 5.4%, while 3.7% is attributable to tobacco use alone [3]. Of the illicit substances, the main drugs used are opiates in European and Asian countries and cocaine in South America and in Africa the preferred drug is cannabis [4].

Youths are a high risk group for the use of substances [5]. Among the youth, substance use is a worldwide epidemic that can impact negatively on health, family, society, and educational and professional life [5–7].

University study is a period when students experience independence and freedom from direct adult and family supervision, self-decision-making, and intense academic pressures, share living quarters with strangers, form new social groups, balance social engagements with academic and other life responsibilities, and may be exposed to normative values valued by the youth culture that differ from parental values [6, 8–12]. These perceived norms motivate the youth to indulge in unhealthy behaviors such as smoking and alcohol and drug use [6, 8]. University students make the transition from the restricted life monitored by parents to a more self-directed life influenced by the university environment [13]. Hence, the risk of substance use is increased in university environments [5].

Recent trends indicate that the use and abuse of substances have dramatically increased worldwide particularly in developing countries [7–9, 14]. Several studies have indicated that substance use is common among students and is becoming increasingly widespread in many African countries [12]. Most countries in sub-Saharan Africa are experiencing rapid economic, social, and cultural transitions which have created favorable conditions for increased and socially disruptive substance use [11]. African studies have indicated that tobacco, alcohol, cannabis, inhalants, tranquilizers, heroin, and cocaine are common in secondary schools and some higher educational institutions [15]. Both alcohol and tobacco are regarded as “gateway drugs” to other substances [5, 7, 12]. Substance use has contributed to the increasing incidence of psychosocial problems among the youth [16]. People with substance use disorder are more likely to die younger and are more likely to have a psychiatric disorder than people without substance use disorder [5, 17].

Although substance use is believed to be a growing problem in Sudan, there is no published data on the magnitude of substance use, particularly among university students, other than tobacco. The aim of this study is to assess the magnitude of substance use and circumstances and factors associated with substance use among university students.

## 2. Materials and Methods

The survey was conducted in February 2014 at a private university in Khartoum State, Sudan. There are thirteen faculties at the university.

All 3,035 full-time undergraduate students at the time of study were eligible to participate in this institution-based survey. The sample size was calculated using the formula *n* = *NZ* ^2^ *P*(1 − *P*)/*d* ^2^(*N* − 1) + *Z* ^2^ *P*(1 − *P*), where *n* is the sample size, *N* is the population size (3,035), *Z* is the statistic for the 95% level of confidence, *P* is the expected prevalence of substance abuse (0.5), and *d* represents precision (0.05). The calculated sample size was 342. Upon considering a potential nonresponse of 15%, the sample size was increased to 393. To conduct a meaningful analysis, the final sample was raised to 500. A quota sampling technique proportional to faculty size, class size, and sex distribution was used to collect the sample. We adopted this technique of sampling due to the sensitive nature of substance use in Sudan. The sample was recruited from the lecture halls. Students were oriented on the purpose of the research and the sampling technique selected. We invited students to participate in the survey to fulfill the quota from each batch.

We defined substance use as the use of any items listed in the questionnaire in lifetime, during the past 12 months or 30 days. Data was collected using a questionnaire designed by the WHO for student drug surveys [18]. The questionnaire was an anonymous self-administered coded questionnaire. The questionnaire was used to collect student background characteristics, type of substance used (tobacco, alcohol, cannabis, heroin, cocaine, amphetamine and amphetamine-type stimulants, inhalants, tranquillizers, and opiates), source of initiation, reasons for substance use, and awareness of negative effects experienced from use of substances. The questionnaire was pretested which aided in uncovering the locally used terms for various substances and enabled questions to be translated into Arabic alongside the English questions. Results of the pretest sample were not included in the final analysis.

Data was analyzed using IBM SPSS version 20. Descriptive frequency analysis was done. Bivariate and multivariate analysis were employed to identify factors associated with the outcome of substance use. Odds ratio with 95% confidence interval was computed to assess the level of association and statistical significance. The level of significance was set at 0.05.

Permission was obtained from the Academic Secretary of the university and deans of the different faculties to recruit the required sample and distribute questionnaires. Students were visited in their classes and oriented on the survey and questionnaire. All recruited students provided verbal informed consent. Students were assured that information would be maintained confidential. The questionnaire did not collect any information that could be used to identify the students. No names or index numbers were collected. Completed questionnaires were dropped by students in a box near the exit of the hall. The university administration has requested to keep the name of the university anonymous in case of publication as it is a private higher educational institute.

## 3. Results

Out of 500 questionnaires distributed, 410 were completed (response rate: 82%). Questionnaires were excluded from analysis if they were incomplete, had inconsistent data, or were not returned.  Table 1 displays the sociodemographic characteristics of the final sample. The sample included more females (70.2%) than males (29.8%). The mean age (SD) was 19.6 (1.9) years. The mean age (SD) for substance users was 19.7 (2) years. The majority of students were from health faculties (87.1%) and of Sudanese nationality (91.2%). The distribution of the sample by study year ranged from 6.8% to 32.4% with the least number of students in year 5 and the greatest number of students in year 3. With regard to accommodation status, 57.6% lived with their parents, 20.2% lived with relatives, 15.3% lived in students' hostel, and 6.8% lived alone in their family's home or rented residence.

The overall prevalence of substance use is 31%. Single substance users constituted 45.7% and multiple substance users 54.3%. The lifetime, past year, and past month prevalence of substance use is displayed in Table 2. Lifetime use of tobacco was reported by 27.8% of students, followed by cannabis (9.3%), alcohol (7.3%), and amphetamines or stimulants use (6.8%). A lesser proportion of students reported lifetime use of tranquilizers (4.9%), solvent inhalants (3.2%), opiates (2.4%), cocaine (1%), and heroin (0.7%). The survey also revealed that past year tobacco use was reported by 18.8% of the students followed by cannabis (7.1%), alcohol (5.6%), and amphetamines or stimulants use (4.6%). Similarly, a lesser proportion of students reported past year use of tranquilizers (3.7%), solvent inhalants (1.5%), opiates (1.5%), heroin (0.7%), and cocaine (0.5%). With regard to substance use within the past 30 days, tobacco, cannabis, alcohol, and stimulant use was reported by 13.7%, 4.9%, 2.7%, and 2.4% of the students, respectively. Fewer students reported tranquilizer, solvent inhalants, heroin, and cocaine use within the past 30 days. More than half of the students initiated substance use at age of 16 or younger for tobacco (52.6%) while eight students initiated inhalants use, six initiated opiates use, three initiated cocaine use, and two initiated heroin use. With regard to alcohol, cannabis, amphetamines, and tranquilizers, most students initiated substance use at the age of 17 or above.

The reasons provided by the students for initiation of substance use are shown in Table 3. Curiosity (33.1%) was the main reason provided for initiation of substance use, followed by pleasure (29.9%), relief of psychological stress (15.7%), and relief of fatigue (11%). Less commonly presented reasons were to treat health disorder, to be sociable, to improve academic performance, to be accepted by others, and to remain awake at night and poor relationship with parents.

Of the adverse effects attributed to substance use (Table 3), health problems were reported by 19.7%, theft or loss of money and valuable items was reported by 19.7%, and poor academic performance was reported by 10.2% of the students. Getting into arguments or fights; relationship problems with parents, friends, and teacher; and accidents and injuries were also reported as adverse effects.

40.9% reported being introduced to substances by friends. Other sources of substance use were family, healthcare personnel, and casual acquaintance. In 38.6%, the source was undocumented (Table 3).

Bivariate analysis between sociodemographic factors and substance use in the past twelve months is shown in Table 4. Being male was significantly associated with substance use (OR (95% CI): 5.6 (3.43, 9.14)). Living with family or relatives was significantly associated with less substance use (OR (95% CI): 0.57 (0.34, 0.95)). Age, nationality, and year of study were not associated with substance use. On multivariate analysis, the odds of substances use were higher among males (OR (95% CI): 5.55 (3.38, 9.17)).

## 4. Discussion

We compared our survey with similar studies with regard to response rates and methodological issues of study population, sample size, sampling technique, and type of university students surveyed. The overall response rate was 82% for this survey. This is in line with other response rates which ranged from 58.3% to 98.7% [6–9, 11, 12, 19]. Our final sample size was 500, but analysis was done for only 410 completed questionnaires. In comparison, other studies have analyzed data for sample sizes ranging from 132 to 1,690 students [5–9, 11, 12, 15, 19–22]. We used a quota technique to recruit students proportional to faculty size, academic year, and sex. Five studies have reportedly used random sampling techniques [5, 7, 9, 11, 15, 21, 22]. Two studies used mixed random and nonrandom techniques [19, 20]. One study used a nonrandom technique for recruiting students and one study did not specify the sampling technique employed [6, 12]. Our survey was conducted among students from medical and nonmedical faculties alike. Several studies did not specify the faculty background of students [7, 9, 11, 15, 20–22]. Four studies were conducted on medical or health sciences students [5, 6, 8, 12]. In most studies, the majority of respondents were males [5, 8, 9, 11, 12, 15, 19, 21]. In our survey, there were a majority of female respondents. This is expected as most students at the university are females. Only one other study reported a female majority and in one study the sex distribution was not mentioned [7, 20].

Substances and drugs abused in Sudan are tobacco, alcohol, cannabis, amphetamine, opiates, barbiturates, khat, and tranquilizers [23]. Tobacco is the most abused substance in Sudan and is consumed in three forms of snuff (locally known as* tomback*), cigarettes, and pipe tobacco.* Tomback* is prepared from locally grown tobacco leaves which are left to dry and then ground to fine powder. It is then moistened with a solution of calcium bicarbonate in water and the moist tobacco is rubbed repeatedly in the hands into very small moist lumps which can then be placed between the upper lip and teeth or lower lip or under the tongue. It is left in the mouth for about 30–40 minutes till the nicotine is absorbed [23]. It is five times cheaper than cigarettes and it is a common belief that it is less harmful [24]. Studies have claimed or suspected that* tomback* is associated with risk of cancer of the oral cavity [25, 26]. Alcohol is banned in Sudan on religious grounds and law and is not practiced openly in public. The only forms of alcohol available in Sudan are* marissa* (native beer) and* araki* (native liquor). Marissa is home-made, produced from fermentation of* dura* (grain), has a low alcohol content (3%–6%), and causes intoxication when consumed in large amounts.* Araki* is produced through primitive methods of distillation of fermented grain, palm dates, or bananas. The liquor produced is adulterated with methanol, aldehydes, and ketones. Sometimes* araki* is taken with* marissa*. Cannabis is locally known as* bango* and is illicitly produced in inaccessible areas or hidden between other crops. The foliage is left to dry, crushed into powder, mixed with honey, and pressed to form a cigar-like head which is wrapped in banana leaves,* dura* leaves, or thick paper. Opiates are smuggled into Sudan while the source of codeine is unprescribed cough syrup. Amphetamines are also smuggled into Sudan [23].

The overall prevalence of substance use for at least one substance was 31% in this study. The prevalence of at least one substance use ranged from 31.1% to 78% in similar studies [7, 9, 11, 12, 15, 21, 22]. There are three plausible explanations for the low overall prevalence for substance use among the students. First, tobacco in Sudan is consumed commonly as* tomback* which is not favored by the students and tobacco is not advertised. Instead, the Ministry of Health of Khartoum State is leading the efforts to limit tobacco consumption by increasing awareness of the health risks of tobacco consumption through media outlets to enforce the antitobacco laws and calling for taxes to be raised on tobacco production. Secondly, the second most consumed substance, alcohol, is banned and not sold publicly in stores but can only be obtained from sites that illegally produce them. Thirdly, the questionable quality of illegally produced alcohol deters students from consuming alcohol. In our survey, single substance users constituted 45.7% and multiple substance users 54.3%. Two studies reported that among their students 62.4% and 64% were single substance users and 35.1% were multiple substance users [11, 21]. Polydrug use places students at a greater risk for alcohol-related motor vehicle accidents, property damage, and troubles with the police [6].

The lifetime prevalence of substance use in our study was in line with reported figures in the literature for tobacco or cigarette smoking (6%–42.8%) and alcohol (6.2%–74.6%), cannabis or cannabis derivatives (2%–95%), amphetamines or stimulants (2.1%–67.9%), opiates (1.3%–8.3%), tranquilizers or sedatives (5.9%–27%), cocaine (0%–10.9%), heroin (0.7%), and solvent inhalants (1.2%–10%) [5, 7–9, 11, 12, 15, 20, 21]. The past year prevalence of substance use of tobacco or cigarette smoking and cannabis or cannabis derivatives, cocaine, and opiates was similar to rates reported in other surveys for tobacco or cigarette smoking (4.3%–22%) and cannabis or cannabis derivatives (1.7%–7.6%), cocaine (0%–0.4%), and opiates (0.4%–8.3%); but rates for alcohol, amphetamines or stimulants, tranquilizer or sedatives, and solvent inhalants were less than rates reported in the literature for alcohol (21.6%–74.6%), amphetamines or stimulants (8.7%–26.7%), tranquilizer or sedatives (13.6%), and solvent inhalants (2.6%) [5, 7–9]. With regard to current use of substance use, the rates were comparable to figures reported in the other studies for tobacco or cigarette smoking (1.7%–27.5%) and cannabis or cannabis derivatives (0.6%–4.2%), cocaine (0%–0.4%), and solvent inhalants (1.9%); but the rates were lower than the rates reported for alcohol (9.3%–58%), amphetamines or stimulants (4.2%–33.3%), opiates (6.1%), and tranquilizers or sedatives (5.3%) [5–9, 11, 12, 15].

Tobacco use is the most common reported substance use by students in this survey. In Sudan, there is no official tobacco control policy specifically targeting adolescents [24]. Smoking is prohibited at the university. In our study, students reported using cannabis more frequently than alcohol, although cannabis is more expensive than alcohol. The reasons could be that cannabis is smoked and shared among peers, whereas alcohol drinking is usually not shared between peers. Another reason could be that the students, being students of a private university, have more money to spend on cannabis than alcohol. Alcohol tends to be consumed by lower socioeconomic classes and is legally banned. Cigarette smoking and cannabis have been reported to be commonly used as a stress coping strategy [7, 27]. Students at the university frequently cope with academic pressures. This may also explain why we found a greater proportion of multiple substance users than single substance users in this survey as compared to other studies [11, 21]. Regarding the low reporting of cocaine and heroin, previous studies have reported low rates of illicit drug use and this may be attributed to either underreporting or a lack of availability of these substances, but only cannabis is reported more frequently compared to other illicit drugs [15]. It has been shown that knowledge of the risks and perceived harmfulness of cocaine and heroin have an inverse relationship with substance use [28]. The low rates of illicit substance use may also reflect the difficulty with obtaining illicit drugs as they are costly. It should also not be forgotten that the possession and use of illicit drugs is regarded as a criminal activity and one that will be penalized under the law in Sudan.

The reasons reported for initiation of substance use by students in this survey were akin to some mentioned in the literature. Factors reported in previous studies for initiation of substance abuse have included academic pressure, low grade point average, to increase academic performance, to keep alert while reading, temptation by peer groups, to relax, desire to experiment, the lure of popularity, availability of substances, high social class, poor mental health, perceived adult drug use, to relieve stress, to improve thinking and sharpness of mind, changing social values, to be accepted by others, to be sociable, lack of religiosity, low self-esteem, to remain awake at night, poverty, to get personal pleasure, academic dissatisfaction, to increase pleasure during sex, to remove boredom and tiredness, to feel high mood, failure in love affair, to cope with problems, poor relationship with parents, family disputes, industrialization, urbanization, globalization, cultism, violence, and conflict ridden cultures [5, 7–9, 11, 12, 15, 19, 21, 22, 29]. Students in this survey reported curiosity and pleasure as the main reasons for initiation of substance use. This probably indicates lack of awareness about substance use and therefore there is a need to improve awareness about the circumstances and hazards of substance use.

Many adolescents have limited awareness of the adverse consequences of substance use [7]. Similarly, the adverse effects attributed to substance use as noted by the students were in line with those mentioned in other studies. These consequences include exposing students to legal repercussions, troubles with police, decreased work, decreased academic performance, engaging in unprotected sex, increased risk of contracting HIV and other sexually transmitted diseases, lung disease, heart disease, gastrointestinal disease, depressive illness, addiction, unintentional injuries, physical fights, illegal behavior, intoxication while working, absenteeism, violent crime, theft, loss of money, indiscipline, relationship problems with parents, relationship problems with friends, problems with teachers, loss of friends and break-up of relations, financial hardships, damage to objects, and other psychiatric disorders such as lethargy, hopelessness, and insomnia, excessive absence from school, and dropping out of university [5, 8, 9, 15, 20, 21, 30–32]. The use of cannabis is associated with development of schizophrenia, anxiety, and depression [7]. The most common adverse effects reported by students in our survey are health problems and theft or loss of money or valuables. The reporting of health problems can be explained by the fact that tobacco is the most consumed substance and most students in this survey are of medical background and thus are aware of the effects of tobacco. The reporting of theft and loss of money or valuable items could be explained by the fact that the second most abused substance is cannabis which is costly. Adverse consequences pose a threat to the health, social and economic fabric of families, communities, and nations [22]. With adequate knowledge about the harmful effects of psychoactive substances, students might be deterred from using some substances [7].

With regard to sources of substance use, several studies have indicated that the sources were mostly peers, family members, relatives, local grocery shops, and chemists [9, 15, 19, 21]. However, not all students would report their source of substance use [21]. The sources of substance use reported by students in our sample were similar to those reported in other surveys. The most common source was peers in this survey. It has been suggested that peers might serve as good role models for substance use intervention program [15].

Factors reported in other studies indicate that male sex and living in hostels were associated with substance use [11, 15, 20–22]. On bivariate analysis of our data, the male sex was significantly associated with greater substance use, indicating that more male students are involved in substance use than females. Males are inherently at risk of substance use and the stress which is associated with present day education is believed to be a predisposing factor for addictive behavior [22]. This may also result from the higher social acceptability of substance use among males [20]. Living with family and relatives is significantly associated with less substance use in our survey. Many students at the university live with their families and many students of expatriate parents live with their relatives. This highlights the protective role of family and close relatives from negative peer influences. The importance of peer influence and its association with tobacco, alcohol, and cannabis was reported previously [17, 24].

The study strength is that it involved students from all faculties. The study limitations include using a quota sample and the findings may not be generalized to the whole university study population. However, the results obtained are in line with other studies. We cannot rule out underreporting of substance use as a result of reporting bias, recall bias, and social desirability bias. Another limitation is that after the pretest we did not consider the issues around reliability and validity of responses.

Our study has revealed that substance use is common and varieties of substances have been used in the past or are used currently at the university. Use of more illicit substances is not alarming. The most common reasons for substance use initiation were curiosity, pleasure, and relief of psychological stress. Commonly reported adverse effects were health problems, theft or loss of money or valuable items, and poor academic performance. The prime source of substances was peers. Being male is the most significant factor for substance use.

## 5. Conclusion 

Substance use is a multietiological universal phenomenon with significant adverse impacts on public health. Early substance dependence has implications in the future of university students and may result in psychiatric disorders; hence, preventing early substance-related problems will reduce the risk of these problems in later adulthood when the magnitude of life stresses is greater [15]. Strategies to control substance use are needed and the university should be more observant. It has been suggested that universities should bring about behavioral change in collaboration with other institutions such as the Ministry of Education and peer educators should be involved in providing education [9]. University students' engagement in many risky health behaviors is a continuation of engagement in such behaviors in high school and therefore prevention programs should be started early [6]. The goal is to reduce the magnitude of substance use. Prevention through education, legislation, and continuous research should be the mainstay of control policy. Health education is paramount. Students' awareness of substances and their harmful consequences should be raised by incorporating health education in the curriculum. Legislation may take the form of an internal university substance use management policy that aims to curb substance use within the university. Methods of relieving academic and social stress to restrict substance use should be preferred and may include extracurricular activities. It has been suggested that interventions designed to help people make use of their time will reduce substance use [15]. Students using substances should be provided with counseling and assisted with treatment options. A special unit within the university may be tasked with provision of this care, particularly for those who develop substance use disorders. The role of parents is a significant factor in curtailing substance use and should be addressed in any potential control strategy. Parents need to be made knowledgeable of the types, risks, and circumstances that lead to substance use among university students and how to provide guidance to their children. Substance use has a wider public health dimension and therefore policies for control of substances should be drafted along with public health interventions at a nationwide level. The nature, circumstances, and factors associated with substance use at the university may differ from other higher educational institutes. Therefore, continuous research within the university and other universities will help in monitoring trends and formulating adequate strategies for control.

## Figures and Tables

**Table 1:** Background characteristics of the students (*n* = 410).

	Frequency	Percentage
Sex		
Male	122	29.8
Female	288	70.2
Faculties		
Health faculties	357	87.1
Nonhealth faculties	53	12.9
Nationality		
Sudanese	374	91.2
Non-Sudanese	36	8.8
Study year		
1	77	18.8
2	133	32.4
3	90	22
4	82	20
5	28	6.8
Accommodation status		
With parents	236	57.6
With relatives	83	20.2
Students' hostel	63	15.4
Living alone in family's home or renting residence	28	6.8

**Table 2:** Lifetime, past year, and current month prevalence of substance use (*n* = 410).

	Frequency	Percentage
Tobacco use		
Lifetime tobacco use	114	27.8
Used tobacco within the last 12 months	77	18.8
Used tobacco within the last 30 days	56	13.7
Alcohol use		
Lifetime alcohol use	30	7.3
Used alcohol within the last 12 months	23	5.6
Used alcohol within the last 30 days	11	2.7
Cannabis use		
Lifetime cannabis use	38	9.3
Used cannabis within the last 12 months	29	7.1
Used cannabis within the last 30 days	20	4.9
Stimulant use		
Lifetime stimulant use	28	6.8
Used stimulant within the last 12 months	19	4.6
Used stimulant within the last 30 days	10	2.4
Tranquilizer use		
Lifetime tranquilizer use	20	4.9
Used tranquilizer within the last 12 months	15	3.7
Used tranquilizer within the last 30 days	13	3.2
Inhalants use		
Lifetime inhalants use	13	3.2
Used inhalants within the last 12 months	6	1.5
Used inhalants within the last 30 days	4	1
Opiates use		
Lifetime opiates use	10	2.4
Used opiates within the last 12 months	6	1.5
Used opiates within the last 30 days	5	1.2
Cocaine use		
Lifetime cocaine use	4	1
Used cocaine within the last 12 months	2	0.5
Used cocaine within the last 30 days	3	0.7
Heroin use		
Lifetime heroin use	3	0.7
Used heroin within the last 12 months	3	0.7
Used heroin within the last 30 days	2	0.5

**Table 3:** Reasons for initiation, adverse effects, and sources of substance use (*n* = 127).

	Frequency	Percentage
Reasons for initiation of substance use		
Curiosity	42	33.1
Pleasure	38	29.9
Relief of psychological stress	20	15.7
Relief of fatigue	14	11
To treat a health disorder	8	6.3
To be sociable	8	6.3
To improve academic performance	8	6.3
To be accepted by others	7	5.5
To remain awake at night	7	5.5
Poor relationship with parents	3	2.4
Reported adverse effects of substance use		
Health problems	25	19.7
Theft or loss of money and valuable items	25	19.7
Poor academic performance	13	10.2
Arguments or fights	12	9.4
Relationship problems with friends	10	7.9
Relationship problems with parents	9	7.1
Accidents or injuries	9	7.1
Problems with teachers	4	3.1
Source of substance use		
Friends	52	40.9
Family	11	8.7
Healthcare personnel	9	7.1
Casual acquaintance	6	4.7
Unknown	49	38.6

**Table 4:** Bivariate analysis between sociodemographic characteristics and substance use in the past 12 months (*n* = 410).

	Yes (*n*/%)	No (*n*/%)	COR (95% CI)	*P* value	AOR (95% CI)	*P* value
Age						
15–19	49 (23)	164 (77)	0.95 (0.60, 1.51)	0.838	1.01 (0.56, 1.84)	0.968
20 or more	47 (23.9)	150 (76.1)	1		1	
Sex						
Male	57 (46.7)	65 (53.3)	5.60 (3.43, 9.14)	0.000	5.55 (3.38, 9.17)	0.000
Female	39 (13.5)	249 (86.5)	1		1	
Nationality						
Sudanese	84 (22.5)	290 (77.5)	0.58 (0.28, 1.21)	0.141	0.85 (0.38, 1.95)	0.695
Non-Sudanese	12 (33.3)	24 (66.7)	1		1	
Type of faculty						
Health	88 (24.6)	269 (75.4)	1.84 (0.84, 4.1)	0.125	1.83 (0.78, 4.31)	0.166
Nonhealth	8 (15.1)	45 (84.9)	1		1	
Year of study						
Years 1-2	50 (52.1)	46 (47.9)	1.05 (0.66, 1.65)	0.847	1.14 (0.63, 2.1)	0.671
Years 3–5	160 (51)	154 (49)	1		1	
Accommodation status						
Living with family or relatives	67 (21)	252 (79)	0.57 (0.34, 0.95)	0.031	0.67 (0.36, 1.21)	0.182
Living with friends or alone	29 (31.9)	62 (68.1)	1		1	
